# A Case of Aneurism in a Chicken1Read before the Pathological Society of Philadelphia.

**Published:** 1900-04

**Authors:** Joseph Sailer, Mazyck P. Ravenel

**Affiliations:** The Pathological Laboratory of the University of Pennsylvania and the Laboratory of the State Live-stock Sanitary Board of Pennsylvania; The Pathological Laboratory of the University of Pennsylvania and the Laboratory of the State Live-stock Sanitary Board of Pennsylvania


### A CASE OF ANEURISM IN A CHICKEN.[1]

BY JOSEPH SAILER, M.D.,

AND

MAZYCK P. RAVENEL, M.D.

*(From the Pathological Laboratory of the University of Pennsylvania and the Laboratory of the State Live-stock Sanitary Board of Pennsylvania.)*

THE specimen presented illustrates an extremely rare condition, this being, as far as we have been able to discover, the first and only case recorded. The bird from which this was taken was sent to Dr. Leonard Pearson, State Veterinarian, for examination, the

[1] Read before the Pathological Society of Philadelphia.

owner fearing that the condition might be contagious and affect others of his flock. We can give no history of the cause or progress of the lesions, as they were discovered by the owner only after the bird had leen killed.

On the left side of the body the skin is dissected up by a large blood clot which overlies the pectoral muscles and coincides closely in size with their area. The skin has ruptured over the lower part, leaving the clot exposed. On the left wing a tumor is seen occupying the outer side of the humerus for the greater part of its length, and showing near the centre an opening in the skin about a half-inch in diameter. On dissection a clot on the breast was found to be continuous with that on the wing, both apparently having resulted from a dilatation, with subsequent rupture of the main artery of the wing about the junction of the subclavian with the axillary.

Sections were cut from three portions—from the middle of the large clot lying along the wing; from the proximal end of this clot, and from the subclavian artery. In the clot itself the interesting features were an enormous number of nuclei, the nuclei of the red blood cells; a very small quantity of fibrin, and a considerable amount of hyaline tissue. The clot was distinctly laminated, and there was some slight evidence in one part of reorganization. The sections were stained by hæmatoxylin and eosin, van Gieson's method, Weigert's fibrin method, and the elastica method. The wall of the artery showed very marked inflammatory change—infiltration with round cells. It was considerably dilated, and rupture had taken place, the blood pouring into the surrounding tissues, pushing under the muscle, which lay close to the artery and forming a false aneurism in this situation. The muscle cells where the blood infringed on them showed extensive alterations. The striations were indistinct, and there was considerable cell infiltration into the intermuscular connective tissue, evidently of some standing. Weigert's fibrin method showed essentially the same changes found in the blood clot, with the addition that a few microörganisms of various types were found in the tissues, evidently a post-mortem invasion. The elastica method failed to reveal any hyperplasia of the elastic tissue in the wall of the artery. The case presents, therefore, some form of degenerative arteritis, possibly secondary to traumatism, with softening of the wall of the artery, its dilatation and subsequent rupture. The large masses of blood over the pectoral region and extending into the wing were hæmatomata and not actual false aneurisms.

